# Mitochondrial transfer and transplantation in tendon, ligament, and enthesis repair: current evidence, mechanistic rationale, and regenerative opportunities

**DOI:** 10.3389/fcell.2026.1927682

**Published:** 2026-09-08

**Authors:** Shilong Meng, Xinlei Fu, Yawei Xu, Zhiliang Peng, Zechen Zhang, Chengjie Wang, Binbin Tang, Xiaolin Shi, Kang Liu

**Affiliations:** 1 The Second School of Clinical Medicine, Zhejiang Chinese Medical University, Hangzhou, Zhejiang, China; 2 Department of Tuina, The First Affiliated Hospital of Zhejiang Chinese Medical University, Hangzhou, Zhejiang, China; 3 Second Department of Orthopedics and Traumatology, The Second Affiliated Hospital of Zhejiang Chinese Medical University, Hangzhou, Zhejiang, China

**Keywords:** enthesis, ligament repair, mitochondrial transfer, mitochondrial transplantation, tendon repair, tendon-to-bone healing

## Abstract

Tendon, ligament, and enthesis injuries often heal through fibrovascular scar rather than restoration of native matrix organization, mechanical function, and graded interface architecture. Mitochondrial dysfunction has emerged as a recurrent feature of these repair failures, linking impaired bioenergetics, oxidative stress, persistent inflammation, cell death, and defective matrix remodeling. In parallel, mitochondria are increasingly recognized as transferable organelles that can move between cells or be delivered therapeutically as isolated mitochondria, mitochondria-rich extracellular vesicles, or engineered mitochondria-based products. This review examines mitochondrial transfer and transplantation as organelle-level mechanisms and therapeutic strategies in tendon, ligament, rotator cuff muscle, and enthesis-related repair. We define key terminology and characterization requirements for verifying mitochondrial identity, integrity, uptake, and function, and synthesize evidence across disease-relevant repair contexts. Direct preclinical support is most developed in tendinopathy/tendon repair and rotator cuff tear-associated muscle degeneration, whereas evidence in anterior cruciate ligament-related repair remains early, and application to enthesis regeneration or tendon-to-bone healing is still largely hypothesis-driven. Further therapeutic development will depend on defined donor sources, product identity, indication-specific potency, tissue retention, dosing strategies, safety assessment, disease-relevant models, and functional endpoints.

## Graphical Abstract

**Figure:**
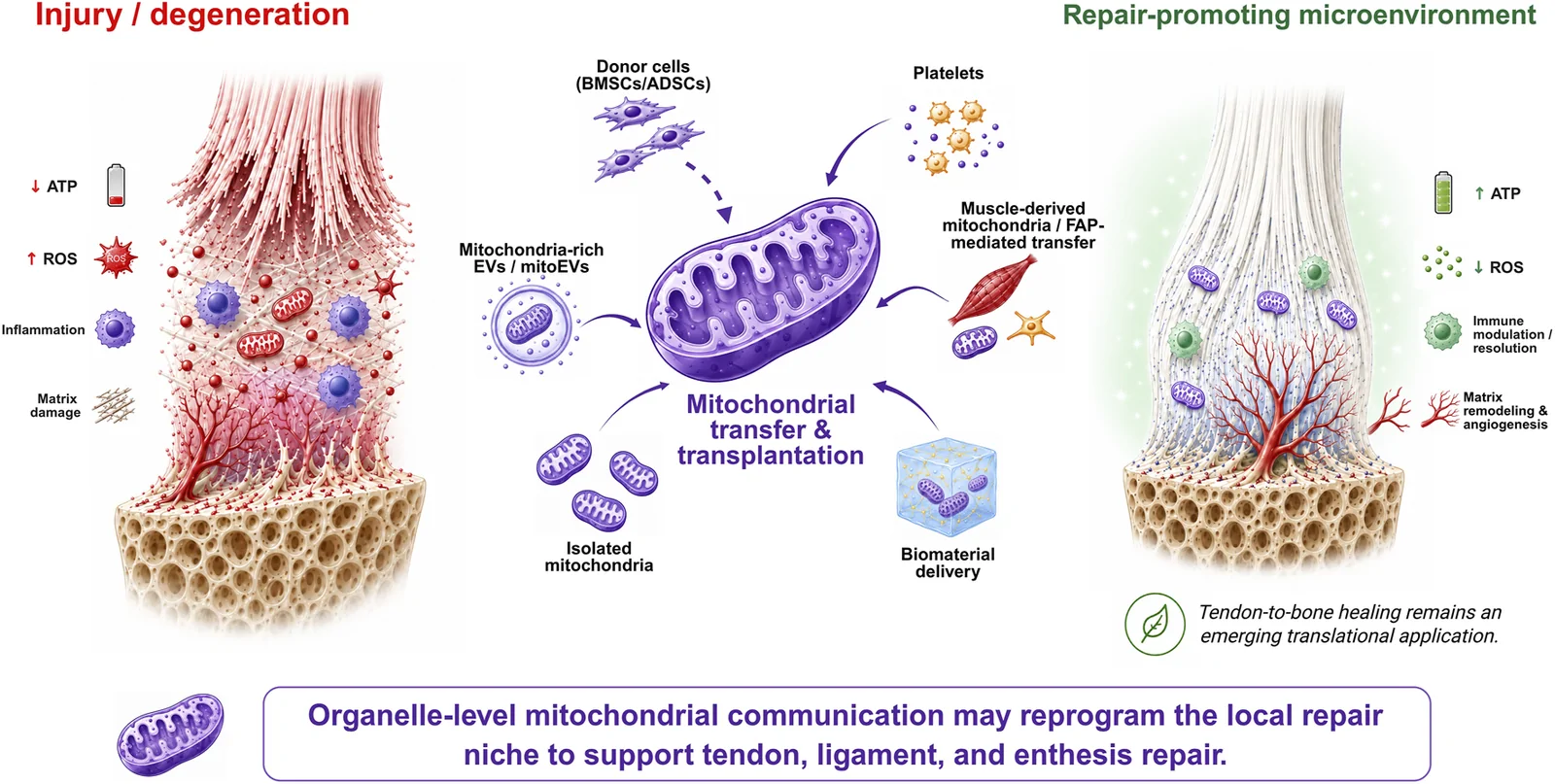

## 1 Introduction

Injuries and degenerative conditions of tendons, ligaments, and entheses remain major challenges in orthopaedic and sports medicine (Ponkilainen et al., 2022; GBD 2021 Other Musculoskeletal Disorders Collaborators, 2023). Tendinopathy, tendon rupture, anterior cruciate ligament (ACL) injury, rotator cuff tear, and failed tendon- or ligament-to-bone healing can cause pain, loss of function, delayed return to activity, and recurrent disability (Hoeffner et al., 2022; Chimenti et al., 2024; Manoso-Hernando et al., 2024; Filbay et al., 2025). Surgery, rehabilitation, biological augmentation, and biomaterial-based strategies have improved treatment, yet durable restoration of native tissue architecture and mechanical function remains uncommon (Zhang et al., 2022; Abdalla et al., 2023; Chen Z. et al., 2023). Healing often produces a fibrovascular scar rather than an organized tendon or ligament matrix. At the enthesis, repair rarely recreates the fibrocartilaginous zone, mineralization gradient, and mechanical integration that normally distribute load across the tendon- or ligament-to-bone interface.

Failed repair is also shaped by the cellular and metabolic state of the local niche. Stromal, immune, and vascular cells, together with fibroadipogenic progenitors and myogenic populations in rotator cuff disease, respond to combined inflammatory, mechanical, and metabolic stress. Persistent maladaptation promotes oxidative stress, chronic inflammation, senescence, matrix catabolism, fibrosis, and fatty infiltration, contributing to chronic tendinopathy, impaired ACL-related repair, rotator cuff tear-associated muscle degeneration, and defective tendon-to-bone healing (Agha et al., 2021; Zhang et al., 2021; Cheng et al., 2024; Leite et al., 2024; Kračun et al., 2025).

Mitochondria sit at the intersection of these stress responses. Beyond adenosine triphosphate (ATP) production, they regulate redox balance, calcium handling, innate immune signaling, apoptosis, and cell fate (Chen W. et al., 2023). In tendon- and ligament-related disorders, mitochondrial dysfunction has been associated with impaired oxidative phosphorylation, excessive reactive oxygen species (ROS), loss of membrane potential, abnormal mitochondrial dynamics, and defective mitophagy (Zhang et al., 2021; Cheng et al., 2024; Kračun et al., 2025; Lui et al., 2022; Pang et al., 2026). These changes can compromise cell survival, extracellular matrix homeostasis, inflammatory resolution, and adaptation to mechanical stress. In rotator cuff tears, mitochondrial impairment is also linked to muscle atrophy, fatty infiltration, fibrosis, and reduced regenerative capacity, with direct implications for repairability and postoperative recovery (Pang et al., 2026; Sahai et al., 2022; Iio et al., 2023; Kusunose et al., 2025).

These observations have expanded the role of mitochondria from intracellular targets to mediators of intercellular communication and potential therapeutic cargo. Functional mitochondria can move between cells through tunneling nanotubes, extracellular vesicles, direct cell–cell contact, cytoplasmic bridges, or uptake of extracellular mitochondria. Mitochondrial transplantation refers to the administration of isolated mitochondria or mitochondria-based products to recipient cells or injured tissues. Unlike conventional mitochondria-targeted pharmacology, these processes can modify donor–recipient interactions at the organelle or mitochondrial-cargo level, with effects that vary according to donor and recipient cell states and the local microenvironment (Li B. et al., 2025).

Orthopaedic evidence remains uneven across repair contexts. Mitochondrial transplantation has improved injured tenocyte function and attenuated inflammation in tendinopathy models, whereas bone marrow mesenchymal stromal cells can transfer mitochondria to damaged tendon cells and promote tendon repair (Lee et al., 2021; Wei et al., 2023). Rotator cuff tear-associated muscle degeneration forms part of the tendon–muscle–enthesis repair unit, and muscle quality strongly influences repairability, functional recovery, and retear risk. In this setting, endogenous mitochondrial transfer involving fibroadipogenic progenitors and myogenic cells, together with therapeutic delivery of mitochondria or mitochondria-rich extracellular vesicles, has been linked to changes in muscle quality, immune remodeling, and regeneration (Chi et al., 2024; Gao et al., 2025; Shi et al., 2025; Wang X. et al., 2025; Milan et al., 2026). Evidence in ACL-related repair remains early, and direct evidence for enthesis regeneration or tendon-to-bone healing is still limited. The enthesis nevertheless provides a biologically relevant setting in which mitochondrial regulation of immune responses, matrix remodeling, and chondrogenic and osteogenic differentiation can be examined.

Existing reviews have focused mainly on mitochondrial dysfunction, oxidative stress, and mitochondrial quality control in musculoskeletal disorders (Zhang et al., 2021; Cheng et al., 2024; Zong et al., 2024; Kubat et al., 2025; Li M. et al., 2025), whereas evidence on mitochondrial transfer and transplantation remains fragmented across studies of tendon, ligament, rotator cuff muscle, extracellular vesicles, and regenerative biomaterials. This review brings these findings into a tendon–ligament–enthesis framework, clarifies terminology and validation criteria, and evaluates the translational relevance of the available evidence.

## 2 Search strategy, study selection, and evidence classification

To define the scope of this review, we searched PubMed, Web of Science Core Collection, and Embase from database inception to 17 June 2026. Search terms covering concepts related to mitochondrial transfer and transplantation were combined with terms related to tendon, ligament, rotator cuff, ACL, enthesis, and tendon-to-bone healing. The core search concept was (“mitochondrial transfer” OR “mitochondria transfer” OR “intercellular mitochondrial transfer” OR “mitochondrial transplantation” OR “mitochondria transplantation” OR “extracellular mitochondria” OR “mitochondria-rich extracellular vesicles” OR “mitoEV” OR “tunneling nanotubes”) AND (“tendon” OR “tendinopathy” OR “tenocyte” OR “ligament” OR “anterior cruciate ligament” OR “ACL” OR “rotator cuff” OR “fibroadipogenic progenitors” OR “enthesis” OR “tendon-to-bone healing”). Database-specific search strategies are provided in Supplementary Table S1.

Records were screened for relevance to the tissue and mitochondrial-transfer scope defined for this review and classified as direct or indirect/contextual evidence. Direct evidence comprised original experimental studies that examined mitochondrial transfer, mitochondrial transplantation, extracellular mitochondria, or mitochondria-rich extracellular vesicles in tendon-, ligament/ACL-, rotator cuff-, enthesis-, or tendon-to-bone-related repair settings. Indirect or contextual evidence included studies of mitochondrial dysfunction, mitochondrial quality control, oxidative stress, immunometabolism, extracellular vesicles, mechanobiology, biomaterial-assisted delivery, or related musculoskeletal regeneration that did not directly test mitochondrial transfer or transplantation in the target repair context. Studies without a clear relationship to the target tissues or repair processes, or without interpretable mitochondrial or repair-related outcomes, were excluded from the evidence synthesis. Reviews, consensus articles, and methodological papers were used to inform terminology, characterization standards, and translational considerations rather than as primary evidence. Reference lists of key articles were also screened manually to identify additional relevant studies.

Evidence was synthesized narratively and organized by repair context: tendinopathy and tendon repair, ligament injury and ACL-related repair, rotator cuff tear-associated muscle degeneration, and enthesis regeneration or tendon-to-bone healing. Evidence maturity was compared descriptively across four dimensions: (1) tissue and disease-context relevance; (2) direct experimental demonstration of mitochondrial transfer, transplantation, or delivery; (3) *in vivo* validation in a repair-relevant model; and (4) repair-related histological, biomechanical, or functional outcomes. Contexts supported by multiple direct, disease-relevant studies with *in vivo* repair outcomes were regarded as relatively more mature; those supported by limited direct preclinical evidence without replicated or durable functional validation were considered early; and contexts lacking direct mitochondrial transfer or transplantation studies were treated as hypothesis-driven. These descriptors were used for narrative comparison across repair contexts and do not constitute a formal GRADE or certainty-of-evidence assessment. Quantitative synthesis was not performed because disease models, mitochondrial sources, delivery routes, and outcome measures were heterogeneous.

## 3 Conceptual framework for mitochondria-based intercellular communication and therapeutic delivery

Mitochondrial transfer and transplantation encompass endogenous organelle exchange, cell-mediated transfer, extracellular mitochondria-containing vesicles, isolated mitochondrial delivery, and engineered mitochondria-based products. Current nomenclature distinguishes endogenous or cell-mediated mitochondrial transfer from therapeutic mitochondrial transplantation and extracellular mitochondria-containing products.

### 3.1 Terminology

Mitochondrial transfer refers to the movement of mitochondria or mitochondrial components from donor cells to recipient cells (Brestoff et al., 2025). This process can occur through direct cell–cell contact, tunneling nanotubes, extracellular vesicles, cytoplasmic bridges, or uptake of extracellular mitochondria. In musculoskeletal repair, relevant examples include mitochondrial transfer from mesenchymal stromal cells to injured tenocytes, from fibroadipogenic progenitors to myogenic cells, and through mitochondria-rich extracellular vesicles acting on immune or muscle-resident cells. The defining feature is not mitochondrial detection alone, but functional delivery capable of altering recipient-cell metabolism, survival, inflammatory phenotype, or differentiation (Brestoff et al., 2025; Al Amir Dache and Thierry, 2023; Cao et al., 2024; Iorio et al., 2024a).

Mitochondrial transplantation refers to the therapeutic administration of isolated mitochondria or mitochondria-based preparations to recipient cells or injured tissues. Unlike endogenous or cell-mediated transfer, transplantation usually involves mitochondrial isolation, quality assessment, dosing, and controlled delivery. Donor mitochondria may be obtained from autologous tissue, allogeneic cells, platelets, stromal cells, skeletal muscle, or engineered cell products. For orthopaedic translation, transplanted mitochondria need to be characterized in terms of purity, viability, immunogenicity, storage stability, delivery route, and tissue retention (Kubat et al., 2025; Li M. et al., 2025; Popov, 2021; Al Amir Dache et al., 2025).

The term extracellular mitochondria encompasses mitochondria or mitochondria-derived materials outside cells, including intact free mitochondria, damaged fragments, mitochondrial DNA (mtDNA), respiratory chain proteins, and mitochondria enclosed within extracellular vesicles. Their biological effects depend on structural integrity and context: intact functional extracellular mitochondria may support bioenergetic rescue after uptake, whereas damaged mitochondrial components can activate innate immune signaling and amplify inflammation. Mitochondria-rich extracellular vesicles (mitoEVs) may contain mitochondrial proteins, mitochondrial DNA, or structurally preserved mitochondria and can serve as routes of intercellular communication or therapeutic delivery. A central distinction is whether these vesicles deliver intact, bioenergetically competent mitochondria or predominantly mitochondrial fragments and associated cargo (Brestoff et al., 2025; Cao et al., 2024; Iorio et al., 2024a; Lou et al., 2025; Wang Y. et al., 2025).

### 3.2 Characterization principles

A recurring challenge in mitochondrial transfer and transplantation studies is distinguishing functional organelle delivery from the detection of mitochondrial labels, fragments, or extracellular debris. Evidence of transfer should not rely on a single fluorescent dye or mitochondrial marker, because dye leakage, vesicle-associated signals, surface adhesion, and residual mitochondrial fragments can all be mistaken for functional mitochondrial delivery (Brestoff et al., 2025; Al Amir Dache et al., 2025).

Characterization should address mitochondrial identity, structural integrity, and bioenergetic competence. Identity can be supported by markers such as TOMM20, COX IV, VDAC, mitochondrial DNA, or mitochondria-selective dyes, but these signals should be interpreted alongside structural and functional evidence. Electron microscopy and complementary mitochondrial membrane and matrix markers can support assessment of structural integrity, whereas membrane potential, ATP production, oxygen consumption, and respiratory complex activity provide functional readouts. Reactive oxygen species generation and calcium handling can further characterize mitochondrial state. For therapeutic preparations, purity, endotoxin burden, storage stability, and reproducibility of mitochondrial yield are also important aspects of product characterization.

Recipient-cell uptake requires separate consideration. Mitochondria detected near or within target cells may reflect adhesion, vesicle uptake, lysosomal degradation, or functional incorporation into the endogenous mitochondrial network. Genetic mitochondrial labeling, donor-specific mitochondrial DNA tracing, species-specific markers, live-cell or three-dimensional imaging, and transfer-blocking experiments can strengthen causal interpretation (Al Amir Dache et al., 2025). Cell fusion represents both an alternative route for donor mitochondrial acquisition and a potential confounder in studies using intact donor cells, because hybrid cells can contain donor-derived mitochondria together with other donor-derived cellular material. Where fusion is biologically plausible, mitochondrial tracing should therefore be complemented by cell-identity or lineage-tracing approaches that distinguish organelle transfer from hybrid-cell formation (Qiao et al., 2024). These considerations are particularly important in tendon-, ligament-, and enthesis-related tissues, where matrix-dense and relatively hypocellular regions can make mitochondrial retention and functional integration difficult to infer from imaging alone.

### 3.3 Mechanisms of mitochondrial transfer and transplantation

Mitochondrial transfer can occur through several routes, including tunneling nanotubes, extracellular vesicles, direct cell–cell contact, cytoplasmic bridges, and uptake of extracellular mitochondria. Tunneling nanotubes are actin-rich membranous structures capable of transporting organelles and signaling cargo between cells. They have been described in multiple injury and disease models and can be promoted by oxidative stress, inflammation, mitochondrial damage, and other stress signals (Al Amir Dache and Thierry, 2023; Ahmad et al., 2014; Liu et al., 2025). In tendon-, ligament-, and enthesis-related repair, they provide a plausible route for stromal-to-resident cell mitochondrial exchange, although direct evidence for tunneling nanotube-mediated transfer in native tendon and enthesis tissues remains limited.

Extracellular vesicle-mediated transfer provides a contact-independent route (Al Amir Dache and Thierry, 2023; Cao et al., 2024; Iorio et al., 2024a). Vesicles may carry mitochondrial proteins, mitochondrial DNA, respiratory components, or mitochondria-like structures, and mitochondria-rich extracellular vesicles may combine paracrine signaling with mitochondrial cargo delivery (Cao et al., 2024; Iorio et al., 2024a; Lou et al., 2025; Wang Y. et al., 2025). Their compatibility with engineering and biomaterial-based delivery may facilitate controlled mitochondrial cargo delivery in orthopaedic applications.

Direct cell–cell contact and cytoplasmic bridges may also support mitochondrial exchange in co-culture systems and local tissue niches, although studies using intact donor cells need to distinguish organelle transfer from cell fusion (Qiao et al., 2024). Mesenchymal stromal cells are among the most studied mitochondrial donor populations and can transfer mitochondria to injured immune, vascular, and musculoskeletal cells (Liu et al., 2025). In tendinopathy models, bone marrow mesenchymal stromal cells transfer mitochondria to damaged tenocytes, improving mitochondrial function and reducing injury-associated cellular dysfunction (Wei et al., 2023).

Mitochondrial transplantation differs from endogenous transfer because isolated mitochondria or mitochondria-based products are delivered directly to cells or tissues. Uptake may involve macropinocytosis, endocytosis, membrane fusion-like processes, or related pathways, depending on product preparation, recipient-cell state, and delivery context. Local injection provides a direct delivery route but may be limited by mitochondrial retention in mechanically loaded tissues. Biomaterial-assisted delivery may improve localization, preserve mitochondrial activity, and prolong exposure at the repair site. Spatial control may be particularly relevant to enthesis and tendon-to-bone healing because of the zonal organization of the tendon- or ligament-to-bone interface (Kubat et al., 2025; Li M. et al., 2025; Popov, 2021).

### 3.4 Donor sources and repair-related recipient cells

The effects of mitochondrial transfer or transplantation depend on the mitochondrial source, delivery context, and metabolic state of recipient cells. Mesenchymal stromal cells are among the most extensively studied donor populations because of their mitochondrial transfer capacity and established relevance to tissue repair. Bone marrow mesenchymal stromal cells can donate mitochondria to injured tenocytes and release mitochondria-rich extracellular vesicles that influence macrophage phenotype and rotator cuff muscle responses (Wei et al., 2023; Gao et al., 2025). Mitochondria isolated from adipose-derived mesenchymal stromal cells have also been tested in chronically injured anterior cruciate ligament cells, with effects on cellular activity and matrix-related repair responses (Lo et al., 2025).

Fibroadipogenic progenitors are particularly relevant to rotator cuff tear-associated muscle degeneration (Agha et al., 2021; Sahai et al., 2022; Iio et al., 2023). These muscle-resident stromal cells can support regeneration, fibrosis, or adipogenesis depending on local cues. Recent studies indicate that fibroadipogenic progenitors can transfer mitochondria to myogenic cells after rotator cuff injury, and that blood-flow restriction may enhance this process while improving muscle regeneration and shoulder function (Chi et al., 2024; Milan et al., 2026). These findings suggest that endogenous mitochondrial transfer may also be responsive to rehabilitation-related stimuli.

Platelets and skeletal muscle provide additional mitochondrial sources of orthopaedic interest. Platelet-derived mitochondria have been evaluated as a local intervention in rotator cuff muscle injury models (Wang X. et al., 2025). Skeletal muscle-derived mitochondria have likewise been explored in rotator cuff-associated muscle repair (Xu et al., 2025), although harvest requirements, mitochondrial yield, and product standardization remain practical considerations.

**FIGURE 1:**
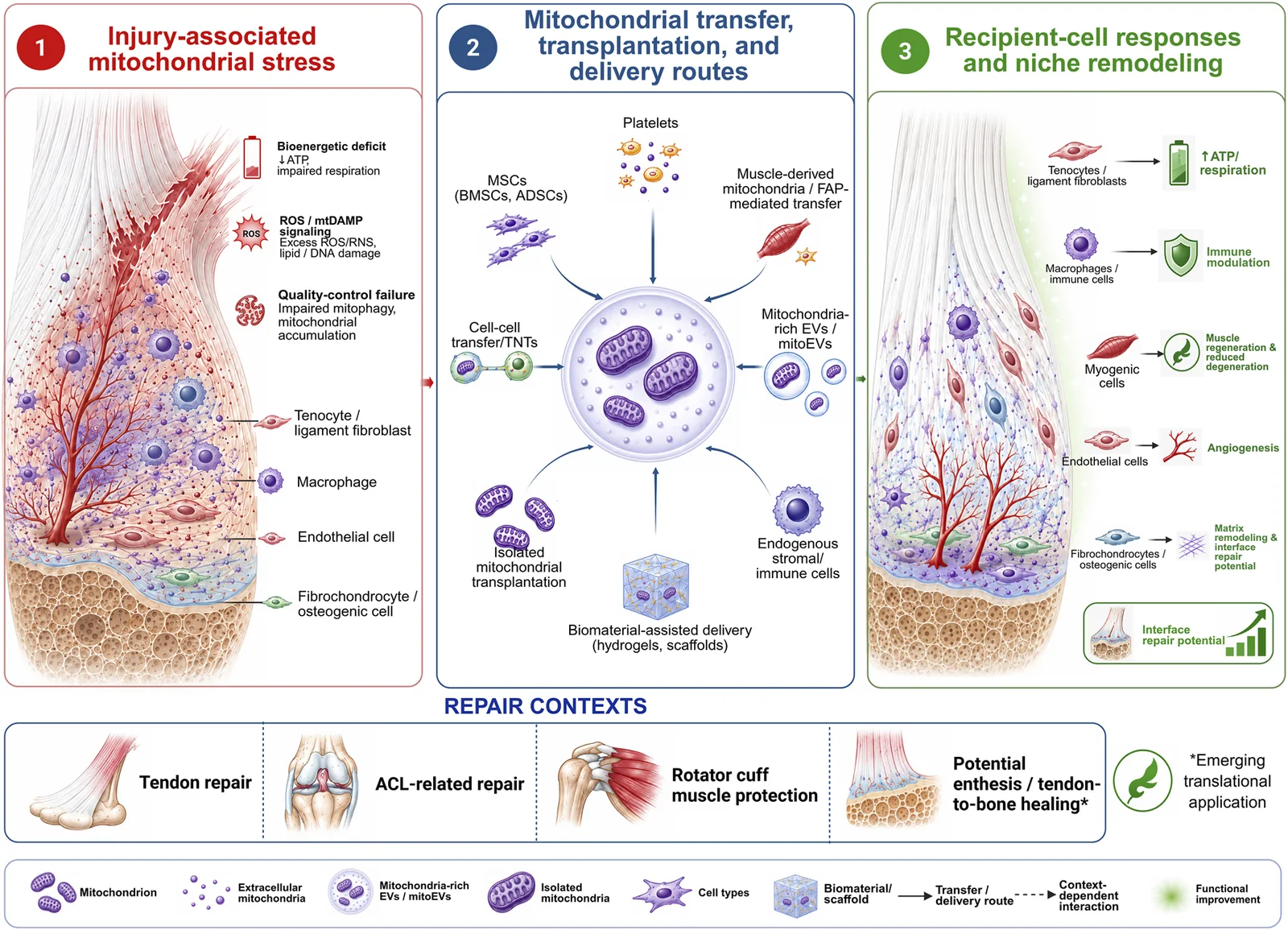
Mitochondrial communication within the tendon–ligament–enthesis repair niche. Panel one depicts injury-associated mitochondrial stress, including impaired bioenergetics, ROS and mitochondrial damage-associated molecular pattern (mtDAMP) signaling, defective mitochondrial quality control, and inflammatory niche remodeling in tendon-, ligament-, rotator cuff-, and enthesis-related tissues. Panel two summarizes mitochondrial transfer, transplantation, and delivery routes, including cell–cell transfer and tunneling nanotubes, extracellular mitochondria, mitochondria-rich extracellular vesicles (mitoEVs), isolated mitochondrial transplantation, biomaterial-assisted delivery, and relevant donor sources. Panel three shows recipient-cell responses and niche remodeling, including metabolic rescue, immune modulation, myogenic regeneration, angiogenesis, matrix remodeling, and tissue integration. The lower row summarizes the repair contexts considered in this review. Direct evidence is strongest in tendinopathy/tendon repair and rotator cuff tear-associated muscle degeneration, whereas application to enthesis regeneration and tendon-to-bone healing remains an emerging translational hypothesis.

Potential recipient populations include tenocytes, tendon stem/progenitor cells, ligament fibroblasts, mesenchymal stromal cells, macrophages, endothelial cells, fibrochondrocytes, osteogenic cells, myogenic cells, and fibroadipogenic progenitors. Their responses to transferred mitochondria are likely to vary with metabolic state, uptake capacity, mechanical environment, and inflammatory context. Donor source, delivery route, recipient-cell state, and repair endpoint therefore need to be considered together when interpreting mitochondrial communication within the tendon–ligament–enthesis repair niche illustrated in Figure 1.

## 4 Mitochondrial dysfunction as a biological bottleneck in tendon–ligament–enthesis repair

Mitochondrial dysfunction links mechanical injury, metabolic stress, inflammation, and defective tissue remodeling in tendon-, ligament-, and enthesis-related disorders. In matrix-dense and mechanically loaded repair environments, impaired mitochondrial adaptation can compromise matrix homeostasis, inflammatory resolution, mechanobiological signaling, and repair-cell function.

### 4.1 Bioenergetic failure and impaired repair-cell function

Repair cells must maintain adequate energy supply while migrating, remodeling matrix, and adapting to changing mechanical demands. In tendons and ligaments, these processes depend in part on mitochondrial oxidative phosphorylation and ATP availability. Injury-related hypoxia, inflammation, and altered mechanical loading can disrupt this energetic balance, impairing cytoskeletal organization, collagen synthesis, matrix remodeling, and cell survival (Zhang et al., 2021; Cheng et al., 2024; Leite et al., 2024).

In tendinopathy, mitochondrial dysfunction has been associated with reduced membrane potential, impaired respiratory chain activity, and altered expression of oxidative phosphorylation-related proteins (Zhang et al., 2021; Cheng et al., 2024; Kračun et al., 2025; Lui et al., 2022; Gao et al., 2024; Lui et al., 2024; Wang et al., 2026). These changes are linked to tenocyte apoptosis, senescence, and loss of extracellular matrix homeostasis. Evidence in ligament injury and ACL-related repair is less direct, but bioenergetic stress may impair ligament fibroblast viability, collagen production, and adaptation to mechanical instability. Direct evidence in enthesis-resident cells remains limited; nevertheless, interface healing requires coordinated remodeling across fibrous, fibrocartilaginous, mineralized, and osseous regions and is therefore likely to place substantial metabolic demands on resident repair cells.

### 4.2 Oxidative stress, mitochondrial damage, and matrix catabolism

At physiological levels, mitochondrial ROS participate in mechanotransduction and matrix remodeling, whereas sustained redox imbalance damages lipids, proteins, DNA, and mitochondrial membranes. In tendon- and ligament-related disorders, excessive ROS can reduce mitochondrial membrane potential, impair respiratory function, activate cell-death pathways, and amplify inflammatory signaling (Kračun et al., 2025; Lui et al., 2022; Zong et al., 2024).

This redox imbalance is closely linked to extracellular matrix breakdown. In tenocytes and ligament fibroblasts, excessive ROS can increase matrix metalloproteinase activity, suppress collagen synthesis, promote inflammatory mediator production, and weaken cell–matrix interactions. In chronic tendinopathy, these changes accompany persistent mechanical stress and catabolic remodeling (Zhang et al., 2021; Cheng et al., 2024; Kračun et al., 2025; Lui et al., 2022; Gao et al., 2024; Wang et al., 2026). Direct evidence specific to enthesis regeneration remains limited, but oxidative damage may also disrupt tendon- or ligament-to-bone healing, which depends on coordinated matrix remodeling, fibrocartilage maturation, mineralization, and bone integration.

### 4.3 Mitochondrial dynamics, mitophagy, and quality control

Mitochondrial fusion, fission, biogenesis, and mitophagy allow repair cells to adapt mitochondrial structure and function to mechanical, oxidative, and inflammatory stress. In tendon- and ligament-related repair, disruption of these quality-control processes can leave injured cells with fragmented or dysfunctional mitochondrial networks, reducing metabolic adaptability and compromising matrix regulation and repair-cell function (Cheng et al., 2024; Zong et al., 2024; Gao et al., 2024; Wang et al., 2026).

In tendinopathy, altered mitochondrial dynamics and defective mitophagy have been linked to tenocyte dysfunction, apoptosis, senescence, and degeneration (Cheng et al., 2024; Gao et al., 2024; Wang et al., 2026). Insufficient removal of damaged mitochondria can sustain ROS production and mitochondrial damage-associated signaling, whereas excessive or maladaptive mitophagy may reduce mitochondrial mass and ATP availability. In rotator cuff tear-associated muscle degeneration, impaired mitochondrial biogenesis and quality control are associated with muscle atrophy, fibrosis, fatty infiltration, and reduced regenerative capacity (Pang et al., 2026; Sahai et al., 2022; Iio et al., 2023; Kusunose et al., 2025).

### 4.4 Mitochondria-mediated inflammation and immunometabolic remodeling

Persistent inflammation can shift tendon-, ligament-, and enthesis-related repair toward matrix degradation, fibrosis, pain, and failed healing. Damaged mitochondria can release mitochondrial DNA, cardiolipin, formyl peptides, and other mitochondrial components that act as damage-associated molecular patterns and sustain innate immune signaling (Zong et al., 2024; Al Amir Dache and Thierry, 2023; Chen et al., 2024).

Mitochondrial metabolism also shapes macrophage behavior. Pro-inflammatory macrophage activation involves glycolytic and mitochondrial metabolic reprogramming that can amplify mitochondrial ROS-dependent inflammatory signaling, whereas reparative activation is associated with greater reliance on oxidative metabolism (Mills et al., 2016; Vats et al., 2006). In tendon and enthesis repair, macrophage state influences matrix remodeling, angiogenesis, fibrosis, and progenitor-cell behavior. Mitochondrial dysfunction may therefore affect repair both through direct effects on resident cells and through changes in the local immunometabolic environment. In tendon-to-bone healing, persistent inflammatory dysregulation can interfere with matrix organization, vascular remodeling, fibrocartilage formation, and mineralized interface maturation (Chen Z. et al., 2023; Al Amir Dache and Thierry, 2023; Morrison et al., 2017; Brestoff et al., 2021; Zhao et al., 2021; Zhao et al., 2026).

### 4.5 Mitochondrial dysfunction in rotator cuff tear-associated muscle degeneration

Rotator cuff tears illustrate that mitochondrial dysfunction within the repair unit is not confined to the torn tendon. After tendon detachment, the supraspinatus and other cuff muscles may develop progressive atrophy, fatty infiltration, fibrosis, and impaired regenerative capacity (Agha et al., 2021; Sahai et al., 2022; Iio et al., 2023; Feuerriegel et al., 2025; Zhang P. et al., 2025). These changes reduce repairability, limit postoperative functional recovery, and increase retear risk. Mitochondrial impairment in this setting is linked to oxidative stress and disturbed lipid metabolism. Experimental rotator cuff injury studies indicate that mitochondrial ROS dysregulation contributes to supraspinatus atrophy, whereas reduced mitochondrial lipid oxidation promotes intramuscular fat accumulation (Pang et al., 2026; Gumucio et al., 2019).

Rotator cuff muscle degeneration arises within a multicellular niche involving fibroadipogenic progenitors, myogenic cells, immune and vascular cells, and muscle fibers. The metabolic state of these populations influences the balance among regeneration, adipogenesis, and fibrosis. Because muscle quality strongly affects tendon repairability and functional recovery, rotator cuff muscle provides an important context for evaluating mitochondrial transfer and transplantation (Agha et al., 2021; Sahai et al., 2022; Iio et al., 2023; Chi et al., 2024).

### 4.6 Rationale for mitochondrial transfer and transplantation

Bioenergetic failure, redox imbalance, defective mitochondrial quality control, and immunometabolic remodeling provide a mechanistic basis for considering mitochondrial transfer and transplantation in tendon-, ligament-, and enthesis-related repair. These approaches aim to restore mitochondrial function by delivering functional mitochondria or mitochondria-containing products to metabolically stressed cells or local repair niches. Such interventions may be particularly relevant in chronically degenerated or severely stressed tissues, where mitochondrial dysfunction is persistent and contributes to impaired repair.

Therapeutic benefit will depend on the quality and functional competence of the mitochondrial source or product, the state of recipient cells, and successful uptake and retention within the target tissue. Short-term mitochondrial detection alone is insufficient to establish repair efficacy; meaningful benefit requires sustained functional effects that translate into improved cellular and tissue-level repair outcomes (Zong et al., 2024; Brestoff et al., 2025; Al Amir Dache and Thierry, 2023; Chen et al., 2024).

## 5 Current evidence across tendon, ligament, and enthesis-related repair

Evidence for mitochondria-based repair differs markedly across orthopaedic contexts. Direct preclinical evidence is strongest in tendinopathy/tendon repair and rotator cuff tear-associated muscle degeneration; ACL-related evidence remains early, whereas enthesis/tendon-to-bone applications are supported mainly by indirect mechanistic evidence. Table 1 summarizes the mitochondrial sources, delivery routes, biological effects, repair outcomes, and evidence maturity across these contexts.

**TABLE 1:** Direct and indirect evidence relevant to mitochondria-based mechanisms and interventions across tendon-, ligament-, rotator cuff-, and enthesis-related repair contexts.

Study	Repair context	Mitochondrial source, product, or process	Recipient cell/tissue and model	Transfer/delivery route and validation	Key mechanism or biological effect	Main repair-related outcomes	Evidence maturity
Lee et al. (2021)	Tendinopathy and tendon repair	Isolated exogenous mitochondria	Damaged tenocytes and rat Achilles tendinopathy model	Direct transplantation of isolated mitochondria; MitoTracker-labeled exogenous mitochondria were visualized in damaged tenocytes and rat Achilles tendon tissue by fluorescence/confocal microscopy	Reduced NF-κB activation, MMP-1 expression, inflammatory mediators, oxidative stress, apoptosis, and fission-associated mitochondrial changes	Restored tendon-related markers, including tenomodulin and collagen I; improved collagen production in injured tendon tissue	Direct preclinical evidence
Wei et al. (2023)	Tendinopathy and tendon repair	Bone marrow mesenchymal stromal cell-derived mitochondria	Damaged tenocytes and collagenase-induced Achilles tendinopathy model	Cell-mediated BMSC-to-tenocyte transfer; donor and recipient mitochondria were differentially labeled with MitoTracker and visualized by fluorescence microscopy. Cytochalasin B markedly reduced transfer and the associated functional rescue; donor mitochondrial signal was also detected in Achilles tendon *in vivo*. Cell fusion was not specifically excluded	Restored mitochondrial membrane potential, oxygen consumption, ATP production, and reduced ROS, inflammatory cytokines, and apoptosis	Improved tenocyte proliferation and increased tendon-related markers, including SCX, TNC, and TNMD; improved tendon repair	Direct preclinical evidence
Li et al. (2026)	Tendon repair	Adipose-derived mesenchymal stromal cell membrane-coated mitochondria	Tendon stromal cells and the Achilles tendon healing model	Engineered cell-free delivery of adipose-derived mesenchymal stromal cell membrane-coated mitochondria (Mito-NPs); mitochondrial preparations and membrane coating were characterized before ROS-responsive hydrogel-assisted delivery to rat Achilles tendon injuries	Metabolic reprogramming and improved mitochondrial targeting/biocompatibility	Restored tendon stromal cell function and promoted Achilles tendon healing	Early direct therapeutic evidence
Chen et al. (2024)	Tendinopathy-related inflammation	Extracellular mitochondrial particles released by tendon cells	Tendon cells and macrophage inflammatory responses under mechanical overload	Stress-induced release of extracellular mitochondrial particles; extracellular particles were characterized by microscopy and mitochondrial markers, including VDAC and TOM20, with mitochondrial labeling used to verify extracellular mitochondrial signals	Extracellular mitochondrial particles acted as inflammatory signals and promoted macrophage chemotaxis and cytokine production	Damaged or stress-derived extracellular mitochondrial particles aggravated tendinopathy-related inflammatory responses	Direct pathological evidence
Lo et al. (2025)	Ligament injury and ACL-related repair	Mitochondria derived from human adipose-derived mesenchymal stromal cells	Chronically injured ACL cells and rabbit ACL partial tear model	Direct transplantation of isolated mitochondria derived from human adipose-derived mesenchymal stromal cells; donor mitochondria and native ACL-cell mitochondria were differentially fluorescently labeled, with confocal imaging confirming donor-mitochondrial uptake in injured ACL cells and fluorescence demonstrating transplanted mitochondria within injured ACL tissue *in vivo*	Improved cellular viability, migration, collagen synthesis, and VEGF expression	Enhanced repair-related activity of chronically injured ACL cells and promoted collagen synthesis *in vivo*	Early direct preclinical evidence
Ma et al. (2023)	Ligament-like stromal and mechanobiological context	Transferred mitochondria in mesenchymal stem cell systems	Human periodontal ligament stem cells under low-stiffness conditions	Transfer of isolated mitochondria from high-stiffness-cultured PDLSCs to low-stiffness PDLSCs; donor mitochondria were fluorescently labeled before transfer, followed by assessment of mitochondrial function and osteogenic rescue	Reversed low-stiffness-induced impairment of osteogenic differentiation	Restored osteogenic differentiation under low-stiffness conditions	Indirect mechanistic support
Luo et al. (2024)	Periodontal ligament stem cell osteogenic differentiation	Bone marrow mesenchymal stromal cell-derived mitochondria	Periodontal ligament stem cells in an inflammatory microenvironment	BMSC-to-PDLSC mitochondrial transfer; transfer was visualized by laser confocal microscopy and quantified by flow cytometry. Cell fusion was not specifically excluded	Enhanced mitochondrial transfer and osteogenic differentiation potential	Increased osteogenic differentiation potential under inflammatory conditions	Indirect mechanistic support
Chi et al. (2024)	Rotator cuff tear-associated muscle degeneration	Endogenous mitochondria from fibroadipogenic progenitor-related systems	Fibroadipogenic progenitors, myogenic cells, and the injured rotator cuff muscle	Endogenous FAP-to-myogenic transfer; mitochondrial dye/flow cytometry was used *in vitro*, and PdgfraCreERT/MitoTag genetic mitochondrial labeling with fluorescence, spinning-disk confocal, and two-photon imaging was used *in vivo*. TdTomato lineage tracing supported exclusion of FAP–myofiber fusion	Linked FAP–myogenic cell crosstalk with mitochondrial communication and muscle regenerative capacity	Identified endogenous mitochondrial transfer as a regulatory mechanism in the injured rotator cuff muscle	Direct endogenous-transfer evidence
Milan et al. (2026)	Rotator cuff tear-associated muscle degeneration and rehabilitation	Endogenous mitochondria mobilized by blood flow restriction therapy	Murine rotator cuff injury model	Blood-flow-restriction-associated endogenous FAP-to-myocyte transfer; Prrx1-Cre/MitoTag reporter mice genetically labeled FAP mitochondria, and green fluorescent protein (GFP)-positive myofibers were quantified histologically in supraspinatus muscle. Cell fusion was not specifically assessed in this study	Blood flow restriction stimulated intercellular mitochondrial transfer and improved regenerative signaling	Improved muscle regeneration and shoulder function in a murine rotator cuff injury model	Direct preclinical evidence; rehabilitation-responsive transfer
Gao et al. (2025)	Rotator cuff tear-associated muscle degeneration	Mitochondria-rich extracellular vesicles from bone marrow mesenchymal stromal cells	Supraspinatus muscle and macrophage-related microenvironment in rat rotator cuff tear model	Mitochondria-rich BMSC-EV delivery; EVs were characterized and tracked in macrophages, while EVs from rhodamine 6G-pretreated BMSCs (Rho-EVs) served as a mitochondrial-function control	Promoted macrophage M2 phenotype conversion and reduced oxidative stress and fibrotic remodeling	Preserved muscle mass, increased muscle fiber cross-sectional area, reduced fibrosis, and improved mitochondrial characteristics	Direct preclinical evidence
Wang H. et al. (2025)	Rotator cuff tear-associated muscle degeneration	Platelet-derived mitochondria	Supraspinatus muscle after rotator cuff tear in rat model	Direct intramuscular administration of isolated platelet-derived mitochondria to the supraspinatus; donor mitochondrial preparations were characterized before delivery, with mitochondrial structure/function and muscle outcomes assessed after treatment	Reduced mitochondrial damage and dysfunction in injured muscle	Attenuated muscle atrophy and fibrosis after rotator cuff tear	Direct preclinical evidence
Shi et al. (2025)	Rotator cuff tear-associated muscle degeneration	Mitochondria isolated from bone marrow mesenchymal stromal cells	Muscle degeneration after rotator cuff tear	Direct intramuscular delivery of isolated BMSC-derived mitochondria; mitochondrial delivery was evaluated in the supraspinatus together with mitochondrial function, ultrastructure, and muscle-repair outcomes	Preserved mitochondrial function, reduced oxidative stress, and promoted angiogenesis-related repair responses	Restrained muscle disuse atrophy and fatty infiltration after rotator cuff tear	Direct preclinical evidence
Xu et al. (2025)	Rotator cuff tear-associated muscle degeneration	Muscle-derived mitochondria	Degenerated rotator cuff-associated muscle in a preclinical model	Direct injection of isolated muscle-derived mitochondria into rotator cuff muscle; purified mitochondrial preparations were characterized and donor mitochondrial distribution was assessed after local delivery together with mitochondrial structure/function and muscle-remodeling outcomes	Improved mitochondrial structure and function, and promoted a more anti-inflammatory local immune environment	Attenuated fibrosis and fatty infiltration, and improved muscle morphological parameters	Direct preclinical evidence
Zhao et al. (2026)	Enthesis and tendon-to-bone healing	No exogenous mitochondrial product; macrophage mitochondrial regulation	Macrophages and tendon–bone healing model	Not applicable — mitochondrial transfer/transplantation was not tested; the study examined endogenous ZEB1–MFN2-dependent mitochondrial dynamics in macrophages	Maintained mitochondrial fission and macrophage efferocytosis; limited inflammation during tendon–bone healing	Improved tendon–bone healing by regulating macrophage-mediated inflammatory resolution	Indirect mechanistic support
Xie et al. (2023)	Enthesis/fibrocartilage preservation in degenerative rotator cuff injury	No exogenous mitochondrial product; chondrocyte mitochondrial regulation	Chondrocytes and fibrocartilage layer in the degenerative rotator cuff injury model	Not applicable — mitochondrial transfer/transplantation was not tested; the study examined SIRT3-dependent mitochondrial homeostasis in chondrocytes/fibrocartilage	Improved chondrocyte mitochondrial homeostasis and alleviated aging-related fibrocartilage degeneration	Promoted healing of degenerative rotator cuff injury and supported fibrocartilage layer preservation	Indirect mechanistic support

Abbreviations: ACL, anterior cruciate ligament; ATP, adenosine triphosphate; BMSC, bone marrow mesenchymal stromal cell; EV, extracellular vesicle; FAP, fibroadipogenic progenitor; MMP-1, matrix metalloproteinase-1; NF-κB, nuclear factor kappa B; PDLSC, periodontal ligament stem cell; ROS, reactive oxygen species; SCX, scleraxis; TNC, tenascin-C; TNMD, tenomodulin; VEGF, vascular endothelial growth factor.

Evidence type in Table 1 describes the relationship of individual studies to mitochondrial transfer or transplantation, including direct therapeutic evidence, endogenous-transfer evidence, pathological extracellular mitochondrial signaling, and indirect mechanistic support. Evidence maturity at the repair-context level was evaluated separately according to tissue relevance, directness of mitochondrial transfer or delivery evidence, *in vivo* validation, and repair-related histological, biomechanical, or functional outcomes. These classifications are descriptive and are not intended as a formal GRADE, or certainty-of-evidence assessment. Transfer/delivery validation summarizes the methods reported in the original studies; unreported validation steps were not inferred. Cell-fusion assessment is reported only where intact donor cells made fusion a plausible alternative explanation.

### 5.1 Tendinopathy and tendon repair

Direct tendon-focused evidence comes primarily from tendinopathy and tendon injury models. Tendon cells operate in a relatively hypovascular and mechanically loaded environment, and studies of tendinopathy have linked mitochondrial bioenergetic failure, redox imbalance, and defective quality control to tenocyte apoptosis, senescence, and loss of matrix homeostasis (Zhang et al., 2021; Cheng et al., 2024; Kračun et al., 2025; Lui et al., 2022; Gao et al., 2024; Wang et al., 2026).

Direct mitochondrial transplantation has shown biological effects in damaged tenocytes and a rat Achilles tendinopathy model. Exogenous mitochondria were delivered to injured tenocytes and detected within Achilles tendon tissue. Treatment increased tendon-related markers, including tenomodulin and collagen I, while reducing NF-κB activation, MMP-1 expression, inflammatory mediator production, oxidative stress, apoptosis, and fission-associated mitochondrial changes (Lee et al., 2021). These findings indicate effects across inflammatory, catabolic, and tenogenic responses in injured tendon tissue.

Cell-mediated mitochondrial transfer provides complementary evidence. Bone marrow mesenchymal stromal cells (BMSCs) transfer mitochondria to damaged tenocytes *in vitro* and improve tendon repair in a collagenase-induced Achilles tendinopathy model. In injured tenocytes, BMSC-derived mitochondrial transfer restored membrane potential, oxygen consumption, and ATP production while reducing ROS, inflammatory cytokines, and apoptosis. It also increased tenocyte proliferation and tendon-related markers, including scleraxis, tenascin-C, and tenomodulin (Wei et al., 2023). Attenuation of the protective effects after transfer inhibition supports mitochondrial donation as one functional component of BMSC-mediated tendon repair.

Engineered mitochondrial delivery has also been explored. Adipose-derived mesenchymal stromal cell membrane-coated mitochondria have been reported to restore tendon stromal cell function through metabolic reprogramming and promote Achilles tendon healing (Li et al., 2026). Membrane coating and biomaterial-assisted delivery may improve localization and preserve mitochondrial activity after local administration, potentially addressing retention limitations in dense and mechanically loaded tendon tissue (Kubat et al., 2025; Li M. et al., 2025; Li et al., 2026).

Mechanical overload can also induce tendon cells to release extracellular mitochondrial particles that promote macrophage chemotaxis and inflammatory cytokine production (Chen et al., 2024). This finding shows that extracellular mitochondrial signaling in tendon can also be pro-inflammatory and depends on the state and context of the released mitochondrial material.

### 5.2 Ligament injury and ACL-related repair

Direct evidence for mitochondrial transfer or transplantation in ligament repair remains limited and centers mainly on ACL-related models. Ligament fibroblasts and ligament-derived cells are relevant recipient populations because successful repair requires cell survival, migration, matrix synthesis, and adaptation to mechanical loading (Lu et al., 2019; Saab et al., 2023; Peng et al., 2025).

The most direct evidence comes from mitochondrial transplantation using mitochondria isolated from human adipose-derived mesenchymal stromal cells in chronically injured ACL cells and a rabbit ACL partial tear model (Lo et al., 2025). Transplanted mitochondria were taken up by injured ACL cells and improved viability, migration, collagen synthesis, and vascular endothelial growth factor expression. *In vivo*, mitochondrial transplantation was associated with increased collagen synthesis and other repair-related responses in the ACL partial tear model (Lo et al., 2025). These findings support cellular and tissue-level biological effects, but biomechanical and durable functional benefits have not yet been established.

Adjacent studies provide mechanistic context rather than ACL-specific evidence. Mitochondrial transfer between bone marrow mesenchymal stromal cells and periodontal ligament stem/progenitor cells enhances osteogenic differentiation in an inflammatory microenvironment, and mitochondrial transfer can reverse low-stiffness-induced impairment of osteogenic differentiation in human mesenchymal stromal cells (Ma et al., 2023; Luo et al., 2024). These findings indicate that mitochondrial exchange can influence osteogenic differentiation under inflammatory or mechanically altered conditions and therefore provide indirect mechanistic support for ligament-related repair. Whether these cellular effects translate into organized ligament repair, biomechanical integrity, ligament–bone integration, and durable joint function remains unresolved.

### 5.3 Rotator cuff tear-associated muscle degeneration: endogenous and therapeutic mitochondrial transfer

Rotator cuff tear-associated muscle degeneration represents one of the most developed preclinical contexts for mitochondrial transfer and transplantation within this review. It is considered here as part of the tendon–muscle–enthesis repair unit because muscle quality influences repairability, postoperative function, and retear risk. Chronic rotator cuff tears are accompanied by muscle atrophy, fatty infiltration, fibrosis, and impaired regeneration, together with metabolic and multicellular remodeling involving fibroadipogenic progenitors, myogenic cells, immune cells, and vascular populations (Pang et al., 2026; Sahai et al., 2022; Iio et al., 2023; Kusunose et al., 2025; Davies et al., 2022).

Endogenous mitochondrial transfer has been demonstrated within injured rotator cuff muscle. Fibroadipogenic progenitors can donate mitochondria to myogenic cells after rotator cuff injury, linking stromal–myogenic communication to muscle regeneration (Chi et al., 2024). Blood-flow restriction has also been reported to enhance intercellular mitochondrial transfer and improve muscle regeneration and shoulder function in a murine rotator cuff injury model (Milan et al., 2026). These findings indicate that rehabilitation-related interventions may modulate endogenous mitochondrial transfer (Milan et al., 2026; Kara et al., 2024; Öberg et al., 2025).

Mitochondria-rich extracellular vesicles have also been evaluated in this setting. Bone marrow stromal cell-derived mitochondria-rich extracellular vesicles mitigated muscle degeneration in a rat rotator cuff tear model, with reparative macrophage-associated responses, preservation of muscle mass and fiber cross-sectional area, reduced fibrosis and oxidative stress, and improved mitochondrial characteristics (Gao et al., 2025). These findings connect mitochondrial cargo delivery with immunometabolic and structural remodeling of the injured muscle niche.

Direct mitochondrial administration has been tested using several donor sources. Platelet-derived mitochondria injected into the supraspinatus after rotator cuff tear attenuated muscle atrophy and fibrosis and reduced mitochondrial damage in a rat model (Wang X. et al., 2025). Mitochondria isolated from bone marrow mesenchymal stromal cells have also been reported to reduce disuse atrophy and fatty infiltration after rotator cuff tear, accompanied by improved mitochondrial function, reduced oxidative stress, and increased angiogenic responses (Shi et al., 2025).

Muscle-derived mitochondria have likewise been evaluated in a preclinical rotator cuff tear model, where treatment improved muscle morphology, reduced fibrosis and fatty infiltration, enhanced mitochondrial structure and function, and shifted the local immune environment toward a more reparative state (Xu et al., 2025).

### 5.4 Potential implications for enthesis and tendon-to-bone healing

Direct evidence that mitochondrial transfer or transplantation promotes enthesis regeneration or tendon-to-bone healing is currently lacking. The enthesis is a spatially graded interface linking tendon or ligament to bone through fibrous, fibrocartilaginous, mineralized, and osseous regions. After injury or surgical repair, this architecture is rarely restored, and healing commonly proceeds through a fibrovascular scar with inferior mechanical integration (Abdalla et al., 2023; Derwin et al., 2018; Zhu et al., 2021; Steltzer et al., 2024).

Current support comes from studies showing that mitochondrial regulation influences processes important to interface healing. The ZEB1–MFN2 axis has been implicated in macrophage efferocytosis and inflammatory control during tendon-to-bone healing, linking mitochondrial state in immune cells to repair quality (Zhao et al., 2026). Mitochondrial regulation of fibrocartilage homeostasis provides another mechanistic connection: activation of SIRT3 in chondrocytes has been reported to reduce aging-related fibrocartilage degeneration and improve healing in degenerative rotator cuff injury (Xie et al., 2023). These findings support a role for mitochondrial function in immune and fibrocartilaginous components of interface repair, but do not directly test mitochondrial transfer or transplantation.

The surgically accessible interface may permit localized delivery of mitochondria or mitochondria-rich extracellular vesicles to resident stromal, immune, vascular, fibrocartilaginous, and osteogenic populations. Biomaterial-assisted systems, including hydrogels, scaffolds, and interface-integrated delivery platforms, could provide spatial control within the tendon- or ligament-to-bone repair environment (Derwin et al., 2018; Zhu et al., 2021; Steltzer et al., 2024; Huang et al., 2020; Zou et al., 2023; Zhang et al., 2024; Li D. et al., 2025; Choi et al., 2023; Song et al., 2023; Qin et al., 2024; Ding et al., 2025; Zhang T. et al., 2025). Direct studies will need to establish mitochondrial uptake, persistence, and repair-related effects in interface-healing models.

**FIGURE 2:**
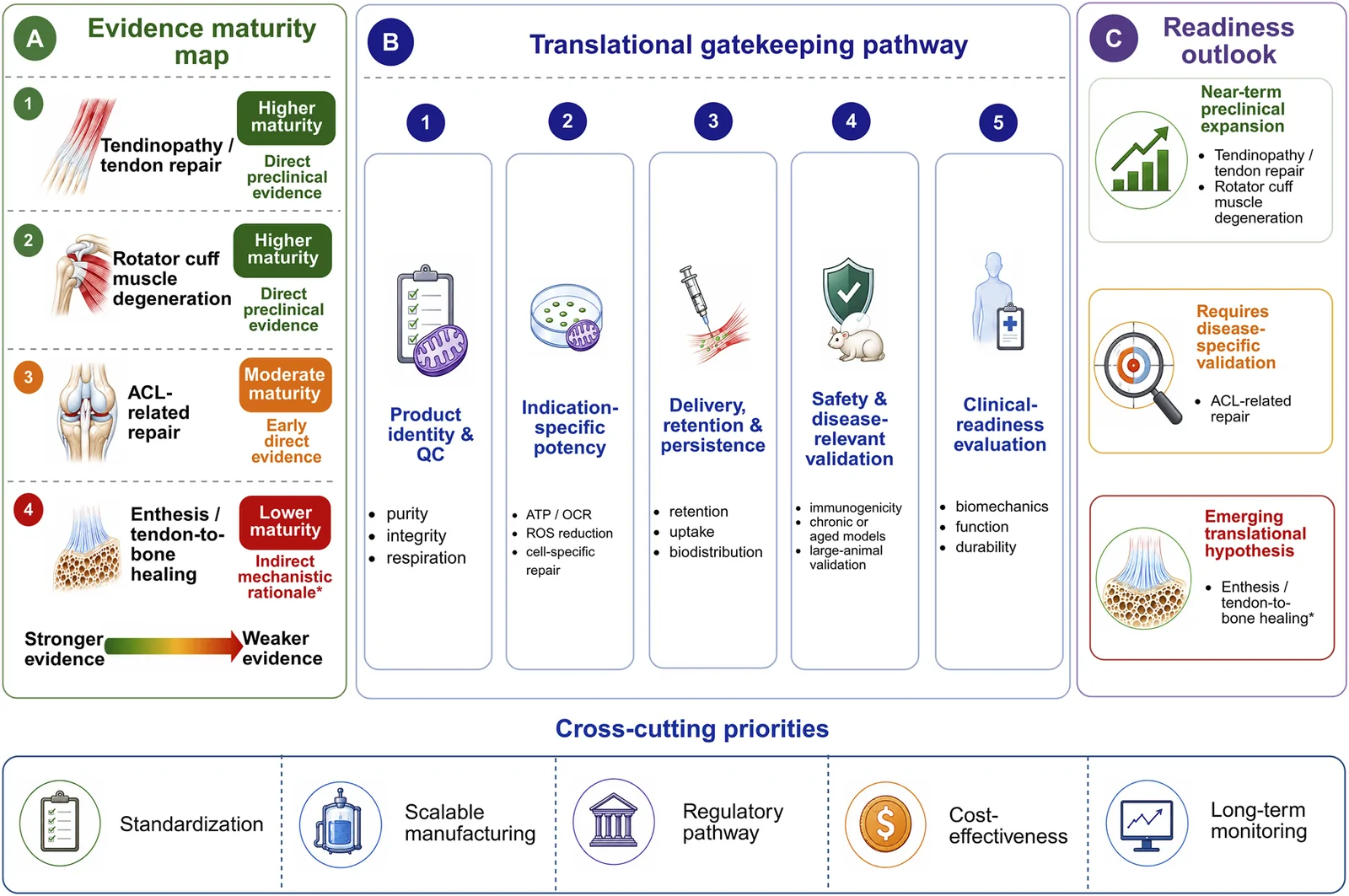
Evidence-to-translation landscape for mitochondria-based strategies in tendon–ligament–enthesis repair. **(A)** Relative evidence maturity differs across repair contexts. Direct preclinical support is most developed in tendinopathy/tendon repair and rotator cuff tear-associated muscle degeneration, whereas ACL-related repair remains early, and enthesis/tendon-to-bone healing is supported mainly by indirect mechanistic evidence. **(B)** Translational gatekeeping requires defined product identity, indication-specific potency, controlled delivery and tissue retention, safety assessment, disease-relevant models, and clinically meaningful endpoints. **(C)** Clinical-readiness evaluation should distinguish mitochondrial uptake from durable tissue-level benefit, including matrix organization, biomechanical performance, muscle quality, inflammatory resolution, and functional recovery. This landscape is descriptive and is not intended as a formal evidence grading system.

The key next step is direct validation showing that mitochondrial transfer or delivery improves tissue organization, biomechanical integration, and functional recovery after tendon-to-bone repair. Until such evidence is available, enthesis applications remain hypothesis-driven. The evidence-to-translation landscape and required validation steps are summarized in Figure 2 *.*

## 6 Discussion: cellular validation, delivery challenges, and therapeutic development of mitochondria-based repair strategies

Translation of mitochondrial transfer and transplantation into musculoskeletal repair requires more than evidence of mitochondrial uptake. Development will depend on defining the delivered product, demonstrating that it retains functional competence at the target site, and linking these effects to repair-relevant tissue outcomes. Product definition, potency, delivery and retention, safety, disease-relevant models, and clinically meaningful endpoints therefore form the main translational considerations summarized in Table 2.

**TABLE 2:** Translational roadmap for mitochondria-based repair strategies in tendon, ligament, and enthesis-related disorders.

Roadmap domain	Key translational question	Key validation criteria	Orthopaedic-specific considerations	Current translational gaps
Product definition and donor source	What is the therapeutic product and where does it come from?	Donor source; isolation method; purity; structural integrity; bioenergetic function; storage condition; delivery vehicle; intended recipient cell or tissue	Source selection may differ across tenocyte-directed therapy, ACL augmentation, rotator cuff muscle protection, and tendon-to-bone interface regeneration	Lack of standardized product definitions; unclear advantages of autologous, allogeneic, platelet-derived, stromal cell-derived, or muscle-derived mitochondria
Quality control and characterization	Is the product structurally intact and functionally active?	Mitochondrial identity; membrane potential; OCR; ATP production; mtDNA content; ROS production; sterility; endotoxin level; non-mitochondrial contamination	Dense connective tissues and local delivery require mitochondria that remain functional after isolation, storage, and implantation	No unified minimum characterization package; limited batch-to-batch comparability
Potency assays	Does the product have indication-specific biological activity?	Tenocyte rescue; collagen synthesis; matrix catabolism; inflammatory mediator suppression; macrophage phenotype; myogenic regeneration; fibrocartilage differentiation; osteogenic and endothelial responses	Tendon repair, ACL-related repair, rotator cuff muscle degeneration, and tendon-to-bone healing require different potency assays	Current assays are often generic and poorly linked to tissue-specific repair mechanisms
Delivery and spatial control	Can mitochondria reach and persist in the target repair microenvironment?	Local retention; biodistribution; uptake route; intracellular fate; lysosomal degradation; functional persistence; release kinetics	Tendons, ligaments, and entheses are dense, poorly vascularized, mechanically loaded, and spatially heterogeneous; tendon-to-bone healing requires zonal delivery	Limited *in vivo* tracking; unclear retention, uptake efficiency, biodistribution, and persistence after local delivery
Dosing and therapeutic timing	What dose, frequency, and treatment window are appropriate?	Mitochondrial quantity; dose-response relationship; single vs. repeated delivery; early vs. delayed treatment; injury-phase-specific effects	Acute injury, chronic degeneration, delayed repair, aging, osteoporosis, fatty infiltration, and inflammatory stress may require different therapeutic windows	Dose metrics, treatment frequency, and therapeutic timing remain poorly standardized
Safety and immunological risk	Could mitochondrial delivery trigger inflammation, off-target effects, or abnormal repair?	Local inflammation; complement activation; off-target distribution; immune compatibility; donor-mtDNA persistence/heteroplasmy; nuclear–mitochondrial compatibility; fibrosis; ectopic mineralization; abnormal angiogenesis; repeated-dose and long-term tissue effects	Damaged mitochondria or mitochondrial components may act as danger signals; allogeneic or engineered products may introduce additional immunological and regulatory risks	Long-term persistence, immunogenicity, genetic compatibility, repeated-dose effects, and disease-context-specific risks remain insufficiently characterized
Disease-relevant preclinical validation	Do models reproduce clinically relevant orthopaedic repair conditions?	Large-animal models; chronic or delayed repair models; aging or osteoporotic backgrounds; clinically relevant reconstruction or repair procedures; rehabilitation-compatible designs	Rotator cuff repair, ACL reconstruction, and tendon-to-bone healing involve mechanical loading, tissue scale, chronic degeneration, and postoperative rehabilitation	Evidence remains dominated by cell and small-animal models; large-animal and disease-relevant validation is limited
Clinically meaningful endpoints	Does treatment produce durable tissue-level and functional repair outcomes?	Biomechanical testing; collagen organization; fibrocartilage regeneration; mineralization gradients; vascular remodeling; mitochondrial persistence; macrophage phenotype; pain-related behavior; gait or shoulder function; retear risk; long-term durability	Endpoint selection depends on the indication: tendon matrix restoration, ligament stability, muscle protection, or tendon-to-bone integration	Many studies rely on short-term cellular, histological, or molecular endpoints without durable functional validation
Integration with existing care	How should mitochondria-based therapy be combined with surgery, biomaterials, orthobiologics, or rehabilitation?	Compatibility with scaffolds, hydrogels, anchors, sutures, extracellular vesicles, platelet-based products, mesenchymal stromal cell-based systems, and rehabilitation protocols	Clinical integration should be evaluated alongside mechanical repair, reconstruction, and rehabilitation	Optimal combination strategies and disease-stage-specific treatment algorithms remain undefined

Abbreviations: ACL, anterior cruciate ligament; ATP, adenosine triphosphate; mtDNA, mitochondrial DNA; OCR, oxygen consumption rate; ROS, reactive oxygen species.

This roadmap summarizes practical translational considerations for mitochondria-based orthopaedic repair.

### 6.1 Donor source selection and product definition

Donor source shapes the biological properties, manufacturability, and potential clinical use of mitochondria-based products. Sources evaluated in orthopaedic and adjacent studies include mesenchymal stromal cells (Wei et al., 2023; Shi et al., 2025; Iorio et al., 2024b; Lo et al., 2025; Li et al., 2026), platelets (Wang X. et al., 2025), skeletal muscle (Xu et al., 2025), and fibroadipogenic progenitors involved in endogenous mitochondrial transfer (Chi et al., 2024; Milan et al., 2026). Mitochondria-rich extracellular vesicles represent a distinct product format in which mitochondrial material is packaged within extracellular vesicles (Gao et al., 2025; Cao et al., 2024; Iorio et al., 2024a; Lou et al., 2025; Wang Y. et al., 2025).

Mesenchymal stromal cell-derived mitochondria have been studied across tendon, ligament, and muscle-related repair contexts, but their properties may vary with donor age, tissue origin, culture conditions, inflammatory exposure, and metabolic state. Platelet-derived mitochondria provide another experimentally tested source, with product characteristics influenced by platelet activation, mitochondrial yield, and preparation method. Skeletal muscle-derived mitochondria have been evaluated in rotator cuff-associated muscle degeneration, although tissue harvest and reproducible mitochondrial recovery remain practical considerations.

Product definition should be source-specific and include the isolation and purification method, structural and functional integrity, storage conditions, delivery vehicle, and intended recipient tissue. Autologous preparations may reduce some immunological concerns but can be affected by the quality of the donor tissue, whereas allogeneic preparations introduce additional requirements for manufacturing consistency, immunological assessment, and regulatory characterization. Source selection may differ among tendon-directed therapy, ACL augmentation, rotator cuff muscle repair, and tendon-to-bone interface applications (Brestoff et al., 2025; Cao et al., 2024; Iorio et al., 2024a; Al Amir Dache et al., 2025; Lou et al., 2025; Wang Y. et al., 2025).

### 6.2 Quality control, potency assays, and reproducibility

Translational quality control should establish mitochondrial identity and purity, structural and bioenergetic competence, microbiological safety, and batch-to-batch reproducibility. Functional measures such as membrane potential, respiratory activity, and ATP production can complement structural characterization, while sterility, endotoxin burden, and non-mitochondrial contamination are important for product release and comparison across studies. For mitochondria-rich extracellular vesicles, product characterization should also establish whether biological activity is associated with intact functional mitochondria, mitochondrial fragments, or other vesicular cargo (Brestoff et al., 2025; Cao et al., 2024; Iorio et al., 2024a; Al Amir Dache et al., 2025; Lou et al., 2025; Wang Y. et al., 2025).

Potency assays should link mitochondrial function to indication-specific repair biology. In tendon repair, this may include mitochondrial rescue together with matrix synthesis or catabolic responses in tenocytes (Lee et al., 2021; Wei et al., 2023). In rotator cuff tear-associated muscle degeneration, relevant readouts include myogenic recovery, mitochondrial metabolism, lipid accumulation, fibrosis, and immunometabolic remodeling (Chi et al., 2024; Gao et al., 2025; Shi et al., 2025; Wang X. et al., 2025; Milan et al., 2026; Xu et al., 2025). For tendon-to-bone healing, potency will ultimately need to be related to interface-specific processes such as inflammatory resolution, fibrocartilage formation, osteogenic remodeling, and tissue integration (Chen Z. et al., 2023; Zhao et al., 2026; Xie et al., 2023). Mitochondrial activity alone is therefore insufficient as a potency measure unless it is linked to repair-relevant cellular or tissue outcomes.

### 6.3 Delivery, tissue retention, and spatial control

Delivery remains a major challenge for mitochondria-based orthopaedic repair. Direct injection has been used in several preclinical models, but locally administered mitochondria may show limited retention, uneven distribution, or loss of function within mechanically active repair sites. Matrix-dense tissues such as tendon, ligament, and the tendon-to-bone interface can further restrict mitochondrial distribution and recipient-cell uptake, making delivery requirements highly dependent on tissue context (Kubat et al., 2025; Li M. et al., 2025; Popov, 2021; Al Amir Dache et al., 2025; Li et al., 2026).

Biomaterial-assisted delivery may improve localization and prolong mitochondrial availability at the repair site (Kubat et al., 2025; Li M. et al., 2025; Li et al., 2026; Song et al., 2023; Wang S. et al., 2025). Hydrogels, scaffolds, and other interface-integrated delivery systems can protect mitochondrial preparations and provide greater spatial control, which may be particularly important at the zonally organized tendon-to-bone interface (Choi et al., 2023; Ding et al., 2025; Zhang T. et al., 2025; Song et al., 2025; Chen et al., 2025; Li X. et al., 2025; Yuan et al., 2025). Cell membrane-coated mitochondria and mitochondria-rich extracellular vesicles provide additional approaches to modulate targeting and uptake, although the delivery vehicle itself may influence mitochondrial function and recipient-cell responses (Cao et al., 2024; Iorio et al., 2024a; Lou et al., 2025; Wang Y. et al., 2025; Li et al., 2026). Evaluation should therefore extend beyond initial uptake to tissue retention, distribution, intracellular fate, and sustained mitochondrial function after delivery.

### 6.4 Dosing, timing, safety, and disease-context specificity

Optimal dose, timing, and dosing frequency have not been established for mitochondria-based orthopaedic repair. Treatment effects are likely to depend on the inflammatory and remodeling state of the target tissue, because mitochondrial delivery can influence immune activity, oxidative stress, matrix remodeling, angiogenesis, and cellular regeneration at different stages of healing. The response also depends on mitochondrial integrity and the local microenvironment. In overloaded tendon, for example, extracellular mitochondrial particles can promote macrophage recruitment and inflammatory cytokine production, illustrating that extracellular mitochondrial material can have distinct effects according to its state and biological context (Lee et al., 2021; Milan et al., 2026; Brestoff et al., 2025; Chen et al., 2024).

Safety is closely linked to product integrity and delivery context. Mitochondrial DNA, cardiolipin, formyl peptides, and other mitochondrial components can activate innate immune signaling when released or exposed inappropriately (Zong et al., 2024; Brestoff et al., 2025). Poorly characterized preparations may therefore provoke inflammatory or immune responses, distribute beyond the intended site, or contribute to maladaptive tissue remodeling. Longer-term evaluation of allogeneic, engineered, or repeatedly administered products should also address donor-mtDNA persistence, heteroplasmy, and nuclear–mitochondrial compatibility (Sercel et al., 2021; Lechuga-Vieco et al., 2022). In cell-mediated approaches, unintended cell fusion remains an additional safety and interpretation concern because it can alter recipient-cell identity beyond selective organelle transfer (Qiao et al., 2024).

Disease context is also likely to influence both efficacy and risk. Mitochondria obtained from aged, inflamed, metabolically stressed, or diseased tissues may differ in functional competence, while chronically degenerated recipient tissues may respond differently from acute injury models. These differences are particularly relevant in chronic rotator cuff degeneration and tendon-to-bone repair, where tissue quality and repair capacity may already be compromised. Malignant models further illustrate the context dependence of mitochondrial transfer, although they do not establish an equivalent tumor-promoting risk in orthopaedic repair (Li B. et al., 2025; Ikeda et al., 2025). Dose-response studies and donor–recipient models should reflect the biological state and intended clinical setting of the target tissue.

### 6.5 Preclinical model design and clinically meaningful endpoints

Most current evidence comes from cell-based studies and small-animal models. These models are useful for investigating mechanism, feasibility, and early safety, but capture only part of the chronic degeneration, tissue scale, mechanical loading, surgical complexity, and rehabilitation encountered clinically. Model selection should therefore match the intended indication. For rotator cuff disease, model relevance depends on capturing muscle degeneration and tendon-to-bone repair together with biomechanical or functional outcomes; for ACL-related repair, ligament–bone integration, joint stability, and biomechanical strength are central (Lo et al., 2025; Lu et al., 2019; Saab et al., 2023; Wong et al., 2025). Models that incorporate clinically relevant tissue context and scale are more informative for translation than acute young-animal injury alone (Kang et al., 2024; Marshall et al., 2024). Aging, osteoporosis, delayed repair, and chronic degeneration are clinically relevant because they alter tissue quality and the metabolic, inflammatory, and regenerative state of the repair environment, which can change treatment response and make acute-injury models less representative of target patients (Kusunose et al., 2025; Derwin et al., 2018; Song et al., 2025; Wang H. et al., 2025).

Endpoint selection should distinguish mitochondrial engagement from repair efficacy. Mitochondrial persistence and immune or metabolic responses can document biological activity, but tissue-level validation requires structural and biomechanical outcomes appropriate to the repair context. At the tendon-to-bone interface, these include collagen organization, fibrocartilage restoration, mineralization, vascular remodeling, tissue integration, and biomechanical strength (Zhao et al., 2026; Derwin et al., 2018; Steltzer et al., 2024; Xie et al., 2023; Song et al., 2023). Functional measures such as gait or shoulder function and durability under loading or rehabilitation provide an additional level of validation (Milan et al., 2026; Lo et al., 2025; Lu et al., 2019; Wu et al., 2026). Standardized reporting of mitochondrial source, dose, route, timing, and outcome definitions will facilitate comparison across studies.

### 6.6 Integration with surgery, rehabilitation, and orthobiologics

Preclinical development should evaluate mitochondria-based interventions within the surgical, biomaterial, orthobiologic, and rehabilitation settings in which they are intended to be used. In rotator cuff repair, a key question is whether mitochondrial interventions can preserve muscle quality and improve the biological environment surrounding tendon repair. At the tendon-to-bone interface, localized mitochondria or mitochondria-rich extracellular vesicles could be combined with biomaterial systems designed for spatially controlled repair (Steltzer et al., 2024; Zhang et al., 2024; Song et al., 2023; Qin et al., 2024; Wu et al., 2026; Zhu et al., 2025). In ACL-related applications, evaluation alongside reconstruction or partial-injury models would allow mitochondrial effects to be assessed within the mechanical environment of ligament repair (Lo et al., 2025; Lu et al., 2019; Saab et al., 2023; Wong et al., 2025). BMSC-derived extracellular vesicles have also been evaluated for tendon-to-bone healing after ACL reconstruction (Li et al., 2022).

Blood-flow restriction provides an example of how rehabilitation may intersect with mitochondrial biology. In a murine rotator cuff injury model, blood-flow restriction enhanced intercellular mitochondrial transfer together with improvements in muscle regeneration and shoulder function (Milan et al., 2026). This finding suggests that rehabilitation-related mechanical and metabolic cues can modulate endogenous mitochondrial communication.

### 6.7 Limitations

The evidence synthesized in this review is predominantly preclinical and heterogeneous in mitochondrial source, product definition, delivery route, injury model, and outcome measures. In addition, the evidence-maturity categories were used as a descriptive framework based on tissue relevance, directness of mitochondrial transfer or delivery evidence, *in vivo* validation, and repair-related outcomes rather than as a formal certainty-of-evidence assessment. These factors limit direct comparison across studies and the strength of clinical inference.

## 7 Conclusion

Mitochondrial transfer and transplantation provide distinct routes for modifying mitochondrial function in tendon-, ligament-, and enthesis-related repair through intercellular organelle exchange or therapeutic mitochondrial delivery. Direct preclinical evidence is strongest in tendinopathy/tendon repair and rotator cuff tear-associated muscle degeneration, whereas ACL-related evidence remains early and enthesis regeneration or tendon-to-bone healing is supported mainly by indirect mechanistic evidence. Further translation will depend on well-characterized mitochondria-based products, effective delivery, disease-relevant models, and demonstration of durable structural, biomechanical, and functional benefit.
