# Supplementary material for: Mitochondrial transfer and transplantation in tendon, ligament, and enthesis repair: current evidence, mechanistic rationale, and regenerative opportunities
Source: Front Cell Dev Biol. 2026 Sep 8;14:1927682. doi: 10.3389/fcell.2026.1927682 (PMC13594376; doi:10.3389/fcell.2026.1927682)
Supplement: Supplementary file 1 [file Table1.docx]

**Supplementary Table S1. Database-specific search strategies**

Search date: June 17, 2026. Search span: database inception to the search date.

Search concept: terms related to mitochondrial transfer, mitochondrial transplantation, extracellular mitochondria, mitochondria-rich extracellular vesicles/mitoEVs, and tunneling nanotubes were combined with terms related to tendon, ligament, rotator cuff, anterior cruciate ligament, enthesis, and tendon- or ligament-to-bone healing. Records were screened for relevance to the review scope.

| **Database/source** | **Field(s)** | **Time span/search date** | **Search strategy** |
| --- | --- | --- | --- |
| PubMed | Title/Abstract | Database inception to June 17, 2026 | (("mitochondrial transfer"[Title/Abstract] OR "mitochondria transfer"[Title/Abstract] OR "intercellular mitochondrial transfer"[Title/Abstract] OR "mitochondrial transplantation"[Title/Abstract] OR "mitochondria transplantation"[Title/Abstract] OR "extracellular mitochondria"[Title/Abstract] OR "mitochondrial extracellular vesicles"[Title/Abstract] OR "mitochondria-rich extracellular vesicles"[Title/Abstract] OR "mitochondria-enriched extracellular vesicles"[Title/Abstract] OR "mitochondria-containing extracellular vesicles"[Title/Abstract] OR mitoEV[Title/Abstract] OR mitoEVs[Title/Abstract] OR "tunneling nanotubes"[Title/Abstract] OR "tunnelling nanotubes"[Title/Abstract]) AND (tendon[Title/Abstract] OR tendons[Title/Abstract] OR tendinopathy[Title/Abstract] OR tenocyte[Title/Abstract] OR "tendon repair"[Title/Abstract] OR ligament[Title/Abstract] OR ligaments[Title/Abstract] OR "ligament repair"[Title/Abstract] OR "anterior cruciate ligament"[Title/Abstract] OR ACL[Title/Abstract] OR "rotator cuff"[Title/Abstract] OR "rotator cuff tear"[Title/Abstract] OR supraspinatus[Title/Abstract] OR "fibroadipogenic progenitor"[Title/Abstract] OR "fibroadipogenic progenitors"[Title/Abstract] OR enthesis[Title/Abstract] OR entheses[Title/Abstract] OR "tendon-to-bone healing"[Title/Abstract] OR "tendon-bone healing"[Title/Abstract] OR "tendon bone healing"[Title/Abstract] OR "ligament-to-bone healing"[Title/Abstract])) |
| Web of Science Core Collection | Topic (TS) | Database inception to June 17, 2026 | TS=(("mitochondrial transfer" OR "mitochondria transfer" OR "intercellular mitochondrial transfer" OR "mitochondrial transplantation" OR "mitochondria transplantation" OR "extracellular mitochondria" OR "mitochondrial extracellular vesicles" OR "mitochondria-rich extracellular vesicles" OR "mitochondria-enriched extracellular vesicles" OR "mitochondria-containing extracellular vesicles" OR mitoEV OR mitoEVs OR "tunneling nanotubes" OR "tunnelling nanotubes") AND (tendon OR tendons OR tendinopathy OR tenocyte OR "tendon repair" OR ligament OR ligaments OR "ligament repair" OR "anterior cruciate ligament" OR ACL OR "rotator cuff" OR "rotator cuff tear" OR supraspinatus OR "fibroadipogenic progenitor" OR "fibroadipogenic progenitors" OR enthesis OR entheses OR "tendon-to-bone healing" OR "tendon-bone healing" OR "tendon bone healing" OR "ligament-to-bone healing")) |
| Embase | Title/Abstract/Keyword (ti, ab, kw) | Database inception to June 17, 2026 | (('mitochondrial transfer':ti,ab,kw OR 'mitochondria transfer':ti,ab,kw OR 'intercellular mitochondrial transfer':ti,ab,kw OR 'mitochondrial transplantation':ti,ab,kw OR 'mitochondria transplantation':ti,ab,kw OR 'extracellular mitochondria':ti,ab,kw OR 'mitochondrial extracellular vesicles':ti,ab,kw OR 'mitochondria-rich extracellular vesicles':ti,ab,kw OR 'mitochondria-enriched extracellular vesicles':ti,ab,kw OR 'mitochondria-containing extracellular vesicles':ti,ab,kw OR mitoev:ti,ab,kw OR mitoevs:ti,ab,kw OR 'tunneling nanotubes':ti,ab,kw OR 'tunnelling nanotubes':ti,ab,kw) AND (tendon:ti,ab,kw OR tendons:ti,ab,kw OR tendinopathy:ti,ab,kw OR tenocyte:ti,ab,kw OR 'tendon repair':ti,ab,kw OR ligament:ti,ab,kw OR ligaments:ti,ab,kw OR 'ligament repair':ti,ab,kw OR 'anterior cruciate ligament':ti,ab,kw OR acl:ti,ab,kw OR 'rotator cuff':ti,ab,kw OR 'rotator cuff tear':ti,ab,kw OR supraspinatus:ti,ab,kw OR 'fibroadipogenic progenitor':ti,ab,kw OR 'fibroadipogenic progenitors':ti,ab,kw OR enthesis:ti,ab,kw OR entheses:ti,ab,kw OR 'tendon-to-bone healing':ti,ab,kw OR 'tendon-bone healing':ti,ab,kw OR 'tendon bone healing':ti,ab,kw OR 'ligament-to-bone healing':ti,ab,kw)) |
| Manual reference screening | Reference lists | Completed after database searches | Reference lists of key original studies, reviews, consensus articles, and methodological papers were screened manually to identify additional relevant records and to clarify terminology, characterization standards, and translational considerations. |

**Notes:** ACL, anterior cruciate ligament; mitoEV, mitochondria-rich extracellular vesicle; TS, topic search in Web of Science. Search strings were adapted to the syntax of each database. Quantitative synthesis was not performed because disease models, mitochondrial sources, delivery routes, and outcome measures were heterogeneous.

**Screening and evidence classification:** Records identified through database searching and manual reference screening were assessed for relevance to the predefined tissue and mitochondrial-transfer scope. Direct evidence was defined as original experimental work that tested mitochondrial transfer, transplantation, extracellular mitochondria, or mitochondria-rich extracellular vesicles in a target repair context. Studies that informed mitochondrial mechanisms, tissue biology, or translational feasibility without directly testing transfer or transplantation in that context were retained as indirect/contextual evidence. Studies without a clear relationship to the target repair tissues or without interpretable mitochondrial or repair-related outcomes were excluded from the evidence synthesis. Evidence maturity was compared descriptively using tissue relevance, directness of mitochondrial transfer/delivery evidence, in vivo validation, and repair-related histological, biomechanical, or functional outcomes.
